# Creating idiometric short-form measures of cognitive appraisal: balancing theory and pragmatics

**DOI:** 10.1186/s41687-021-00317-x

**Published:** 2021-07-13

**Authors:** Carolyn E. Schwartz, Roland B. Stark, Bruce D. Rapkin

**Affiliations:** 1 DeltaQuest Foundation, Inc., 31 Mitchell Road, Concord, MA 01742 USA; 2 Departments of Medicine and Orthopaedic Surgery, Tufts University Medical School, Boston, MA USA; 3 Department of Epidemiology and Population Health, Albert Einstein College of Medicine, Bronx, NY USA

**Keywords:** Appraisal, Response shift, Individual differences, Cognitive, Measurement, Idiometric

## Abstract

**Background:**

The Rapkin and Schwartz appraisal theory and measure provided a path toward documenting response-shift effects and describing individual differences in ways of thinking about quality of life (QOL) that distinguished people in different circumstances. Recent work developed and validated the QOL Appraisal Profile_version 2_ (QOLAP_v2_), an 85-item measure that taps response-shift-detection domains of Frame of Reference, Standards of Comparison, Sampling of Experience, and Combinatory Algorithm. Recent theoretical work proposed that appraisal measurement constitutes a new class of measurement (idiometric), distinct from psychometric and clinimetric. To validate an idiometric measure, one would document that its items *reflect* different circumstances and population characteristics, and *explain* variance in QOL. The present work sought to develop idiometric short-forms of the QOLAP_v2_ item bank by examining which items were most informative, retaining the appraisal-domain structure.

**Methods:**

This secondary analysis (*n* = 1481) included chronically-ill patients and their caregivers from a longitudinal web-based survey (mean follow-up 16.6 months). Data included the QOLAP_v2_, the Center for Disease Control Healthy Days Core Module, the PROMIS-10 Global Health, and demographic/medical variables. Appraisal items were measured at baseline (relevant to understanding cognitive appraisal processes); and with change scores (sensitive to response-shift effects). Multivariate analysis of covariance examined what demographic and health-status change variables were *reflected* by each of 85 appraisal items (in five sets), as dependent variables, and other demographic/medical variables. Multiple linear regression examined how appraisal items *explained* variance in global physical- and mental-health change, after covariate adjustment. A tally summarized item performance across all five sets of cross-sectional and longitudinal analyses.

**Results:**

The vast majority (i.e., 80%) of the QOLAP_v2_ items performed well across the analyses presented. Using a relatively strict criterion of explaining meaningful variance across 60% of analyses, one would retain 68 items. A more lenient criterion (40%) would retain 71.

**Conclusions:**

The present study provides heuristics to support investigators’ creating ‘discretionary’ QOLAP_v2_ short-forms to fit their study aim and amplifying individual differences in the cognitive processes underlying QOL. This approach enables adapting the measure to the study population, as per the expectation that respondent populations differ in the predominant cognitive processes used.

**Supplementary Information:**

The online version contains supplementary material available at 10.1186/s41687-021-00317-x.

## Background

The study of response-shift phenomena has been facilitated over the past 25 years by response-shift theory [1–3] and related methodological development [4–11]. While many response-shift-detection methods rely only on quantitative analysis of patterns suggestive of response shift (e.g., measurement invariance [5, 11]), there are also several methods that combine qualitative and quantitative characterization of response-shift effects (e.g., Schedule for the Evaluation of Individual Quality of Life [12], Patient-Generated Index [13], Quality of Life Appraisal Profile (QOLAP) [2]). Rapkin and Schwartz introduced in 2004 a more testable version of the Sprangers and Schwartz response-shift theoretical model [1] and the QOLAP [2, 14]. The QOLAP combined open- and closed-ended questions to characterize individual differences in four domains necessary for characterizing response-shift effects: Frame of Reference comprised of Quality of Life (QOL) Definition and Goal Delineation; Standards of Comparison; Sampling of Experience; and Combinatory Algorithm [2]. Building on Tourangeau’s [15] theoretical and empirical work on the psychology of survey response, the Rapkin and Schwartz appraisal theory and initial measure provided a path toward not only documenting response-shift effects [2, 3, 16], but also describing the differences in ways of thinking about QOL and patterns of emphasis that distinguished people who fared better or worse with chronic medical conditions such as human immunodeficiency virus [7], multiple sclerosis [17], bladder cancer [18], and spinal disorders [19].

The four appraisal domains are elements of the *contingent true score* described in Schwartz and Rapkin [14]. In this formulation, any rating of a QOL item reflects a latent QOL “true score” that is contingent upon processes of QOL appraisal. Thus, numerical agreement in QOL scores does not guarantee that respondents arrived at their responses in the same way [14]. Two respondents may both rate themselves as doing poorly but base their conclusion on different observations [14]. Understanding these differences in appraisal fosters better communication between patient and caregiver and increases our ability to predict or explain QOL scores [14]. Characterizing changes in appraisal enables detection of response-shift effects. Specifically, changes in Standards of Comparison reflect recalibration; changes in Sampling of Experience and/or Combinatory Algorithm reflect reprioritization; and changes in Frame of Reference reflect reconceptualization [2].

In an effort to make the collection of QOL-appraisal data more feasible, Rapkin and Schwartz developed and validated two closed-ended measures [20, 21]. The QOLAP version 2 (QOLAP_v2_) [20] includes 85 items and taps the four abovementioned domains. This measure has the advantage of retaining the theoretical foundation and having closed-ended items that are less resource-intensive than the original mixed-method QOLAP. The Brief Appraisal Inventory (BAI) [21] was created to be a parsimonious appraisal measure that emphasized the most prominent patterns found in our appraisal research to date [22–24], but it did not aim to represent equally the four appraisal domains. While one can evaluate general response-shift effects using the BAI, one cannot characterize the three aspects of response shift (recalibration, reprioritization, reconceptualization [2]) because the four appraisal domains are not fully captured by the BAI [3].

### Appraisal measures are idiometric

Since the original 2004 papers [2, 14], our group has come to a better understanding of the nature of appraisal measurement [25]. Specifically, appraisal tools are *idiometric*. Accordingly, the items aim to reflect a broad range of possible attitudes/behaviors that inter-correlate in expected and meaningful ways, and which differ across individuals/groups and within individuals/groups over time. With this goal, appraisal measures contrast with psychometric measures, which aim to assess a universal latent construct. Appraisal measures also contrast with those of clinimetric measures, which aim to identify an underlying clinical phenomenon [25]. For a full description of the theoretical distinctions, statistical implications, and clinical applications that characterize idiometric versus psychometric and clinimetric measures, the interested reader is referred to [25].

### Reflecting versus explaining variance

Besides the separate ways in which appraisal at baseline and appraisal change scores can address QOL comparisons and response-shift detection, respectively, we draw another distinction. Ways of appraising QOL can both *reflect* and *explain* variance in other constructs. Seen in the role of reflecting variance, appraisal can reveal both universal and circumstantial aspects of experience [25]. In other words, both antecedents (stable characteristics of the individual) and catalysts (health-state changes, life events, etc.). can affect appraisal, revealing the universal and contextual aspects of experience. This role is shown most naturally when appraisal is used as a dependent variable in a model. For example, we would expect alignment of personal goals with cultural norms for age and gender (universal aspect), while there may be differences in the importance of accomplishing work goals among people facing disruptive life events, such as the diagnosis of new illness or becoming a grandparent (circumstantial).

Seen in the role of explaining variance, appraisal can provide an important context for comparing QOL scores across individuals, both cross-sectionally and over time. As noted above, shared appraisal processes underlie a contingent true score, enabling comparison across individuals [14]. Appraisal change over time serves a separate function, that of revealing response-shift effects in QOL discrepancy scores [2, 3]. In this role of explaining variance, appraisal would most naturally serve as an independent variable in a model. It may also moderate the effect of other independent variables. For example, the emotional impact of multiple chronic health conditions is generally known (universal), but its impact on a particular individual may depend on whether his/her Standards of Comparison focus on others with similar health conditions versus how they were when they were younger (circumstantial).

### Criteria for creating a short-form of an idiometric measure

*An idiometric measure would be considered effective if it either reflected variance or explained variance related to individual differences in circumstances and experience.* Accordingly, creating a short form of an appraisal tool would thus proceed differently than it would for a psychometric tool (e.g., focus on unidimensionality, internal consistency, consistent structure across samples, etc.) or for a clinimetric tool (e.g., focus on relevance to diagnosing and/or distinguishing clinical phenomena) [25]. Such creation would seek to retain a broad universe of content by sampling from an appraisal item bank that encompasses the relevant multidimensional concepts. We would, for example, hypothesize that a measure of appraisal should look different for people at different developmental levels or with different socioeconomic contexts (i.e., different challenges to bear). It should look different for people who are working vs. retired, of different health status or level of wealth. We would select a parsimonious set of items on the basis of reflecting or explaining variance across QOL constructs and/or sociodemographic characteristics.

### Magnitude of effect

In terms of effect-size (ES) metrics, we would consider important even those ES considered “small” [26]. Such “small” effects contribute to a better understanding of what matters to an individual’s QOL at one point in time and over time. ES provides a generic metric of the magnitude of the effect, rather than a score or change score that is specific to the reported measure. ES is also less impacted by sample size, whereas *p*-values can be highly significant for negligibly small effects due to their sensitivity to sample size. Finally, small ES can amplify response-shift-adjusted estimates of treatment-related change [27], such that what was a small unadjusted treatment effect can become a medium ES when response-shift effects are considered – even small ones.

### Toward an idiometric appraisal short form

In summary, an idiometric measure should reflect and/or explain variance related to individual differences in circumstances and experience. Item selection for a short-form variant should focus on both such criteria, so that the eventual measure is useful not only for describing the relevant cognitive processes in a cross-sectional study, for example, but also for detecting response-shift effects in longitudinal data. Triangulating on the “best” items for an appraisal measure must rely on multiple criteria.

## Methods

### Aim

The present work thus sought to understand which QOLAP_v2_ items were most informative so that one or more short-forms of the QOLAP_v2_ could be developed, retaining the appraisal-domain structure.

### Sample

This secondary analysis utilized data collected from Rare Patient Voice and WhatNext panels, with a heterogeneous grouping of chronic health conditions. These panel-research organizations recruit patients and caregivers representing over 200 diagnoses, by attending patient-advocacy conferences. This recruitment approach ensures that participants have or care for someone with the specified diagnosis. Participants sign up to be included in panels to facilitate research on the disease in question, participation for which they may be paid honoraria if resources permit. Eligible participants were patients or caregivers of someone with a chronic medical condition, age 18 years or older, and able to complete an online questionnaire. The present academic study was unfunded and thus unable to pay honoraria.

### Design and setting

A web-based survey was administered twice (baseline, follow-up) using the Health Insurance Portability and Accountability Act (HIPAA)-compliant, secure Alchemer engine (www.alchemer.com). (See [28] for full description of baseline methods, and [16] for follow-up and selection-bias analyses.) The study was reviewed and approved by the New England Review Board (NEIRB#15-254), and all participants provided informed consent.

### Measures

The QOLAP_v2_ [20] is an 85-item measure of cognitive-appraisal processes invoked when answering QOL measures. Four domains are assessed using closed-ended rating-scale items with a response scale ranging from “not at all like me” (1) to “very much like me” (5) or “not applicable/decline” (− 99). The Frame of Reference domain queries how the individual thinks about QOL [QOL Definition (20 items)] *and* what personal goals matter most to their QOL [Goal Delineation (33 items)]. Sampling of Experience (14 items) queries the individual’s heuristics or criteria for responding to QOL measures. Standards of Comparison (9 items) queries to whom or what the individual compares him/herself to when thinking about QOL. Combinatory Algorithm (9 items) assesses the individual’s patterns of emphasis, i.e., what aspects of QOL are considered more salient or more important than others. (The interested reader can contact the authors for a copy of the measure.)

Outcome Measures included the *Center for Disease Control Healthy Days Core Module* [29] and the Patient-Reported Outcome Measurement Information System (*PROMIS)* Global Short Form (*PROMIS-10)*. The third item of the former asks how many days of the past 30 the respondent’s poor physical or mental health kept them from doing their usual activities of daily living (ADL), such as self-care, work, or recreation. The PROMIS-10 yielded scores for global physical and global mental health [30].

Demographic / medical variables included in the analyses included age, gender, ethnicity, race, cohabitation/marital status, with whom the person lives, employment status, disability status (i.e., binary variable indicating if disabled from employment), and difficulty paying bills [31]. Number of comorbidities was measured with the *Self-Administered Comorbidity Questionnaire* [32]. ZIP code was used to characterize in which region of the contiguous US the participant lived [33].

### Statistical analysis

Two sets of analyses aimed at identifying items that 1) *reflected* diverse perspectives and/or 2) *explained* variability in health outcomes in the present sample. In both sets of analyses, we used appraisal items both at baseline and in change scores. Baseline appraisal is relevant to understanding cognitive processes at a given point in time, whereas change-in-appraisal scores are needed to detect response-shift effects per se [2, 3]. We also focused on three separate indicators of QOL change: change in activities of daily living, global physical health, and global mental health. Using QOL change indicators as dependent variables is central to response-shift studies. According to appraisal theory [2], response shift is inferred when change in appraisal explains the discrepancy between expected and observed change in QOL. This discrepancy is often operationalized as the residuals in a “standard QOL model,” i.e., predicting QOL change after adjusting for sociodemographic and other characteristics [3]. As an independent variable, QOL change can help one see to what extent an appraisal item might account for variance in the QOL item. These two complementary approaches help to gauge the relevance of an appraisal item to individual circumstances.

#### Reflecting variability

To understand what demographic and health-status change variables were reflected by appraisal items, six separate multivariate multiple regressions used baseline appraisal or change-in-appraisal items by domain as dependent variables. Predictors included alternate indicators of QOL change (change in activities of daily living, global physical and mental health) and demographic characteristics (region, gender, age, comorbidities, difficulty paying bills, whether working, whether retired, whether disabled). We used a multivariate analysis of covariance (MANCOVA) procedure because predictors included both continuous and categorical variables and we wanted to evaluate all appraisal items within each domain in a single procedure. Even though we were not focusing on group differences, we still retain the label “MANCOVA” for ease of distinguishing from the multiple regression models described below.

#### Explaining variability

To understand how appraisal items explain variance in QOL outcomes, four separate multiple regression models were conducted for each appraisal item as an independent variable. In addition to baseline and change-in-appraisal in separate models, independent variables included selected demographic covariates. Dependent variables were global physical and mental health. Via interactions, these covariates helped account for the expectation that a measure of appraisal will have different slopes for people at different developmental levels (age), different health status (comorbidity burden), or different socioeconomic contexts (difficulty paying bills). To avoid model overspecification, we chose these sociodemographic characteristics based on exploratory analyses of their ability to predict with at least a small effect size on average. We did not want to impede our ability to detect meaningful differences in change by using baseline scores as covariates. Generally speaking, baseline and follow-up QOL scores are often highly correlated and may share some of the same predictors, such as appraisal items. We did not want to rule out meaningful items due to this statistical artifact.

#### Tally of results

To summarize results across all 50 models, we noted those items that explained enough variance to constitute at least a small ES. A small ES for a dependent variable in a MANCOVA model would require an eta^2^ or R^2^ greater than 0.02 [26, 34]. A small ES for an individual predictor in a regression model would require an increment to eta^2^ or R^2^ of greater than 0.01. Effect sizes (ES) of small, medium, and large magnitude using Cohen’s criteria were shown using conditional formatting in tables via light, medium, and fully saturated highlighting.

##### Statistical assumptions and power

The focus of the above-mentioned analyses is to evaluate the reflective and explanatory value of a large set of items, with an eye toward selecting a subset for inclusion in a recommended short-form. Parametric statistical methods are used and treat the rating-scale (ordinal) items as continuous. Although statistical orthodoxy suggests that ordinal data should be analyzed with non-parametric methods [35, 36], for parametric analytical methods, such as regression or analysis of variance, one commonly encounters judgments such as “individual rating items with numerical response formats at least five categories in length may generally be treated as continuous data.” [37] (Also see [38].) Even moderate violations of parametric assumptions (i.e., of normal distributions, interval level of measurement, and homogeneity of variances) have been shown to have little or no effect on substantive conclusions in most instances [34]. Further, the testing of homogeneity of variances for methods such as regression or analysis of variance is generally performed when two or more samples are being compared [39], which is not the case in the present work [39]. Finally, we rely on the magnitude of total model explained variance, rather than the magnitude, direction, or *p*-values for predictors’ coefficients, thereby further reducing the risk of inappropriate inference. Consequently, we utilize these parametric approaches and do not specifically test the assumptions of the 50 tested models. Readers interested in examining variable distributions are referred to Supplemental Table 1. Although as mentioned we do not focus on *p*-values, this study is, incidentally, more than well-powered to detect a small ES in the context of a multivariable linear model with eight covariates (α = 0.05; N required = 757; actual *N* = 1391 [26]). The focus of analyses is only on ES rather than p-value, due to the exploratory nature of the research question and the large number of analyses conducted.

##### Software

Data analyses were implemented using SPSS version 26 [40] and the R software [41].

## Results

### Sample

The study’s analytic sample included 1481 people, comprising 1212 patients, 227 caregivers, and 42 patient-caregivers. The sample represented 212 distinct diagnoses, ranging from rare conditions such as Marfan Syndrome (*n* = 2), which occurs in 1 in 5000 people, to more common medical illnesses such as Breast Cancer (*n* = 246) and Multiple Sclerosis (*n* = 217). Mean follow-up was 16.9 months (standard deviation [SD] = 1.7; range = 13.5–25.7). Table 1 provides the reported sociodemographic characteristics and comorbidities of this heterogeneous sample. Supplemental Table 1 provides descriptive information about the items and QOL summary scores. Interested readers are referred to an in-depth treatment of appraisal inter-item correlations across many populations, which provided the foundation for the “idiometric” distinction [25].

**Table 1:** Person-Level Demographic Characteristics (*N* = 1481)

Variable		
Role	Patient	82%
	Caregiver	15%
	Both	3%
	Missing	0%
Follow-Up Time (Months)	Mean (SD)	16.9 (1.7)
Age	Mean (SD)	49.9 (13.2)
Age at diagnosis	Mean (SD)	41.1 (17.2)
Had help completing questionnaire		2%
Gender	Male	14%
	Female	86%
	Missing	0%
Number of comorbidities out of a possible 15	0	4%
	1	11%
	2	14%
	3	18%
	4	17%
	5	14%
	6	11%
	7 or more	12%
	Missing	0%
Marital Status	Never Married	14%
	Married	61%
	Cohabitation/ Domestic Partnership	7%
	Separated	2%
	Divorced	12%
	Widowed	4%
	Missing	0%
Ethnicity (%)	Not Hispanic or Latino	93%
	Hispanic or Latino	4%
	Missing	3%
Race (%)	Black or African American	4%
	White	93%
	Other	1%
	Missing	2%
Income (%)	Less than $15,000	8%
	$15,001 to $30,000	14%
	$30,001 to $50,000	17%
	$50,001 to $100,000	29%
	$100,001 to $150,000	13%
	$150,001 to 200,000	5%
	Over $200,000	3%
	Missing	12%
Region (named regions are in USA)	East North Central	18%
	East South Central	5%
	Middle Atlantic	11%
	Mountain	7%
	New England	6%
	Pacific	14%
	South Atlantic	16%
	West North Central	7%
	West South Central	9%
	Non-Contiguous	1%
	Other	7%
Difficulty Paying Bills	Not difficult at all	36%
	Slightly difficult	23%
	Somewhat difficult	18%
	Very difficult	10%
	Extremely difficult	10%
	Missing	4%
Employment Status	Employed	48%
	Unemployed	11%
	Retired	13%
	Disabled Due To Medical Condition	27%
	Missing	1%
Work Complexity (past or present)	Mean (SD), 1–5 scale	3.3 (1.1)
Education	Some high school	1%
	High school diploma/GED	20%
	Technical or trade school degree	18%
	Bachelor’s degree	33%
	Graduate or professional degree	26%
	Missing	2%
Mother’s Education	Some high school	13%
	High school diploma/GED	45%
	Technical or trade school degree	14%
	Bachelor’s degree	17%
	Graduate or professional degree	9%
	Missing	2%
Father’s Education	Some high school	14%
	High school diploma/GED	37%
	Technical or trade school degree	14%
	Bachelor’s degree	16%
	Graduate or professional degree	14%
	Missing	5%

Some sets of percentages may not add up to 100% due to rounding

*GED* = General Educational Development (i.e., high-school equivalency test), *SD* = standard deviation

### Selection biases and missing data

Previous reported analyses characterized selection biases in the longitudinal analytic sample [16]. Participants retained in the study were slightly older, less likely to be caregivers, more likely to have arthritis, and less likely to have an ulcer or stomach disease compared to those not retained. They were also more likely to be non-Hispanic, White, and slightly more educated; and to be/have been engaged in an occupation requiring extensive preparation. Out of 1481 participants, 0–7% were missing a value for any given variable included in a model, other than the appraisal item. In the case of region, where 7% were missing values, we categorized such cases as “unknown” and incorporated that as a new category in analysis. The end result was a listwise N of 1391 for MANCOVAs and regressions, which meant 6.1% of cases were excluded in either type of procedure.

### Items that reflected or explained more variability

Supplemental Tables 2 and 3 show results of preliminary exploratory analyses aimed at narrowing down a set of eight demographic predictors for the regression models. These tables supported retaining age, comorbidities, and difficulty paying bills.

Tables 2, 3, 4, 5 and 6 detail the results of MANCOVAs and regression models evaluating baseline and change items for each of the QOLAP_v2_ domains. Models evaluating baseline appraisal are relevant to understanding cognitive appraisal processes at a given point in time, whereas those addressing change-in-appraisal scores are more relevant to detecting response-shift effects per se*.* The value of keeping the three dependent variables of change in ADL, global physical and mental health separate is apparent in looking at patterns across Tables 2, 3, 4, 5 and 6. While some appraisal items perform similarly across outcomes, there are frequently differences between global mental health and the two other dependent variables. Further, there are distinct differences in explained variance when comparing models evaluating baseline appraisal versus appraisal change. This varied information is then summarized in a tally across QOLAP v2 domains (Table 7). We will summarize below the number of items showing small, medium, and large ES for baseline variability and sensitivity to response-shift effects (i.e., to change scores) for each domain. Supplementary Table 4 provides 95% confidence intervals for the explained variance estimates (i.e., eta^2^ and R^2)^ of the MANCOVA and regression models.

**Table 2:**
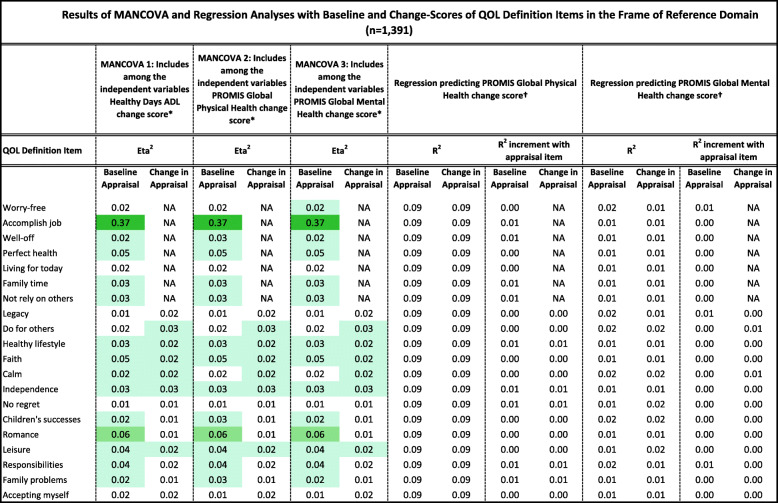
Results of MANCOVA and Regression Analyses with Baseline and Change-Scores of QOL Definition Items in the Frame of Reference Domain (*n* = 1391)

Conditional formatting indicates the magnitude of the effect size (ES), with the least saturated highlighting showing small ES, the middle saturation indicating a medium ES, and the most saturated indicating a large ES

* Dependent variables: QOL Definition Items; Independent Variables: region, gender, age, comorbidities, difficulty paying bills, whether working, whether retired, whether disabled

† Independent Variables: QOL Definition item; 3 covariates -- age, comorbidities, difficulty paying bills; & QOL Definition item’s interactions with the covariates

**Table 3:**
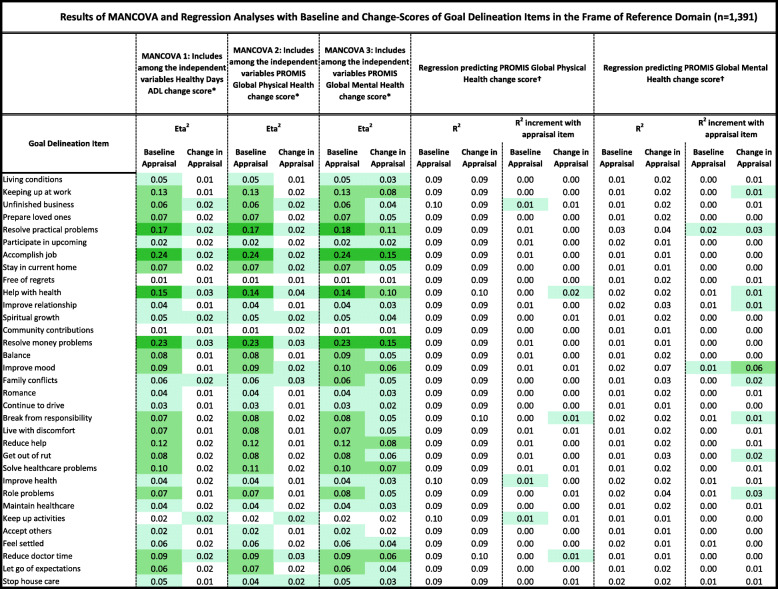
Results of MANCOVA and Regression Analyses with Baseline and Change-Scores of Goal Delineation Items in the Frame of Reference Domain (*n* = 1391)

Conditional formatting indicates the magnitude of the effect size (ES), with the least saturated highlighting showing small ES, the middle saturation indicating a medium ES, and the most saturated indicating a

* Dependent variables: Goal Delineation Items; Independent Variables: region, gender, age, comorbidities, difficulty paying bills, whether working, whether retired, whether disabled

† Independent Variables: Goal Delineation item; 3 covariates of age, comorbidities, difficulty paying bills; & Goal Delineation item’s interactions with the covariates

**Table 4:**
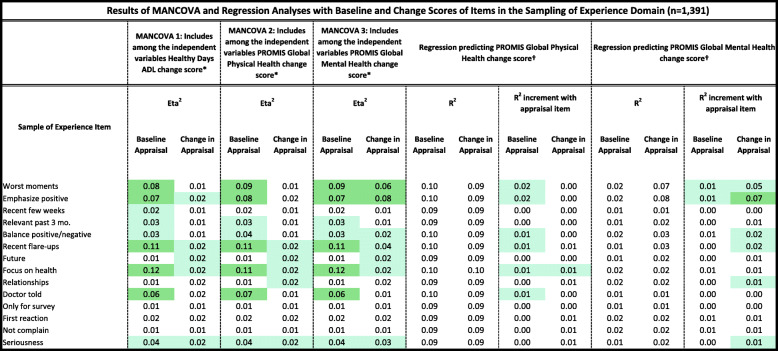
Results of MANCOVA and Regression Analyses with Baseline and Change Scores of Items in the Sampling of Experience Domain (*n* = 1391)

Conditional formatting indicates the magnitude of the effect size (ES), with the least saturated highlighting showing small ES, the middle saturation indicating a medium ES, and the most saturated indicating a large ES

* Dependent variables: Sampling of Experience Items; Independent Variables: region, gender, age, comorbidities, difficulty paying bills, whether working, whether retired, whether disabled

† Independent Variables: Sampling of Experience item; 3 covariates of age, comorbidities, difficulty paying bills; & Sampling of Experience item’s interactions with the covariates

**Table 5:**
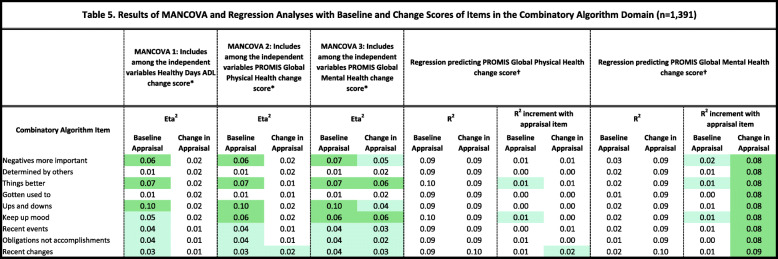
Results of MANCOVA and Regression Analyses with Baseline and Change Scores of Items in the Combinatory Algorithm Domain (*n* = 1391)

Conditional formatting indicates the magnitude of the effect size (ES), with the least saturated highlighting showing small ES, the middle saturation indicating a medium ES, and the most saturated indicating a large ES

* Dependent variables: Combinatory Algorithm Items; Independent Variables: region, gender, age, comorbidities, difficulty paying bills, whether working, whether retired, whether disabled

† Independent Variables: Combinatory Algorithm item; 3 covariates of age, comorbidities, difficulty paying bills; & Combinatory Algorithm item’s interactions with the covariates

**Table 6:**
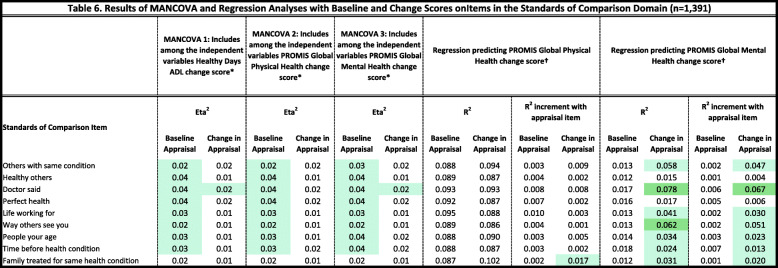
Results of MANCOVA and Regression Analyses with Baseline and Change Scores onItems in the Standards of Comparison Domain (*n* = 1391)

Conditional formatting indicates the magnitude of the effect size (ES), with the least saturated highlighting showing small ES, the middle saturation indicating a medium ES, and the most saturated indicating a large ES

* Dependent variables: Standards of Comparison Items; Independent Variables: region, gender, age, comorbidities, difficulty paying bills, whether working, whether retired, whether disabled

† Independent Variables: Standards of Comparison item; 3 covariates of age, comorbidities, difficulty paying bills; & Standards of Comparison item’s interactions with the covariates

**Table 7:**
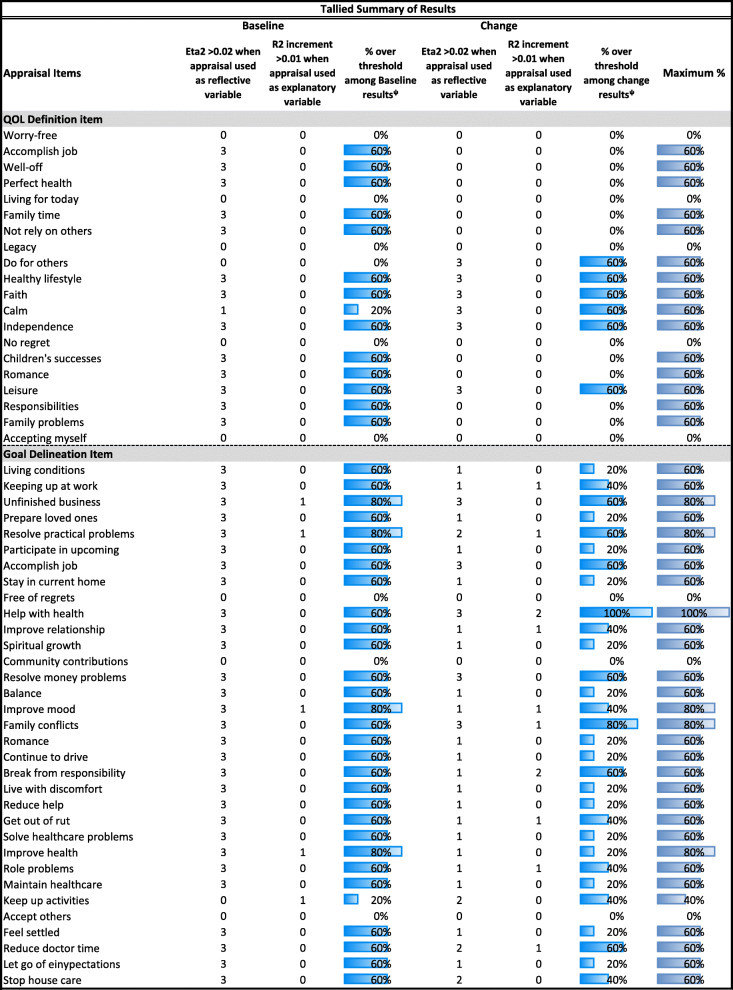

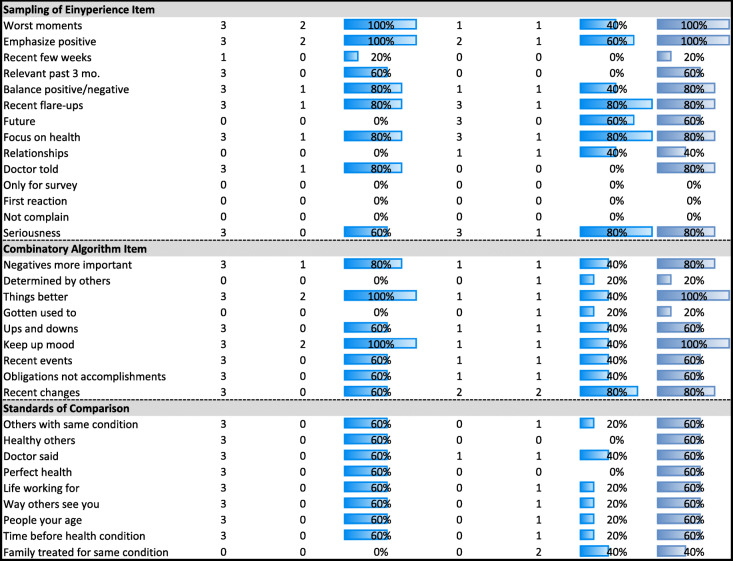
Tallied Summary of Results

#### QOL-definition items

Fifteen of the 20 baseline items and six of the 20 change scores explained at least a small ES of variance in the MANCOVAs (Table 2). Two baseline items and no change scores stood out in terms of explaining medium or large ESs. None of the items, however, explained substantial unique variance in the regression models predicting global physical or mental health.

#### Goal-delineation items

Thirty of the 33 baseline items and 29 of the 33 change scores explained at least a small ES of variance in the MANCOVAs (Table 3). Nineteen baseline items and four change scores stood out in terms of explaining medium or large ESs. Five of the baseline items and none of the change scores explained a small ES of unique variance in the regression models predicting global physical or mental health.

#### Sampling-of-experience items

Nine of the 14 baseline items and nine of the 14 change scores explained at least a small ES of variance in the MANCOVAs (Table 4). Five baseline items and no change scores stood out in terms of explaining medium or large ESs. Six of the baseline items and their change scores explained a small ES of unique variance in the regression models predicting global physical or mental health.

#### Combinatory-algorithm items

All seven of baseline items and all seven of the change scores explained at least a small ES of variance in the MANCOVAs (Table 5). Four baseline items and no change scores explained medium or large ESs. Three of the baseline items and their change scores explained a small ES of unique variance in the regression models predicting global physical or mental health.

#### Standards-of-comparison items

Eight of the nine baseline items and seven of the nine change scores explained at least a small ES of variance in the MANCOVAs (Table 6). No baseline items or change scores explained medium or large ESs. No baseline items and seven change scores explained at least a small ES of unique variance in the regression models (five small ES, two medium ES).

#### Tally of item performance across QOLAP_v2_ domains

Comparing across the above results, Table 7 provides a tally of each appraisal item’s performance across the five baseline and five change-score analyses. This tally shows separately, for all domains and items, in what proportion of the analyses each item showed at least a small ES in MANCOVA analyses (Eta^2^ when appraisal used as a reflective variable) and in regression analyses (R^2^ when appraisal used as an explanatory variable). The column on the far right shows the maximum of the baseline- and change-score performances, because both the baseline and change scores are independently important (i.e., the former help to understand individual differences cross-sectionally; the latter are sensitive to response-shift effects). This tally suggests that the vast majority (i.e., 80%) of the QOLAP_v2_ items perform well across the analyses presented (i.e., explain at least a small ES). Using a criterion of evidence of value in at least 60% of analyses (i.e., 3 out of 5 models), our results support retaining 15 out of 20 QOL Definition items; 29 of 33 Goal Delineation Items; 9 of 14 Sampling-of-Experience items; 7 of 9 Combinatory-Algorithm items; and 8 of 9 Standards-of-Comparison items.

## Discussion

The present study provides intelligence about the cross-sectional and longitudinal informativeness of the QOLAP_v2_ items across domains. For many investigators, an 85-item measure is prohibitively long. Results of the present study can thus be useful for creating ‘discretionary’ short forms rather than a one-size-fits-all short-form solution. Given the fact that appraisal items are used in MANCOVAs as dependent variables, we would expect explained variance associated with a given item would be greater in the MANCOVAs than in the regressions because the former each focus on multiple predictors explaining an appraisal item. In contrast, in the regressions, appraisal items were more narrowly considered because they were tested only for their predictive power in accounting for a single dependent variable.

Since this idiometric measure [25] is intended to amplify individual differences in the cognitive processes underlying QOL, and respondent populations are expected to differ in the range and predominant cognitive processes used, it would make sense that short forms of the QOLAP_v2_ would be chosen to vary by study population. Patient populations differ in the inter-relationships of appraisal items, varying as a function of population differences in circumstances, background, and experiences [25].

An important distinction in this idiometric validation study is that the end result is not a “static short form” or a “computerized adaptive test” but rather an heuristic. Results of the present study provide heuristics to support investigators’ liberty in selecting the best brief subset of items to fit their study aim. Those that performed less well would not be retained. In this context of idiometric measurement, investigators might select items that appear relevant to their study population on the basis of not only item performance shown herein and inter-item correlations shown elsewhere [25], but also by considering the unique circumstances of their study sample, research questions, intervention, etc. that they wish to amplify via the appraisal items. The 85 items included in the QOLAP_v2_ represent research done on a broad range of patient groups using mixed methods and comprehensive study to generate the closed-ended items [16, 17, 19, 20, 22, 23, 25, 28]. We thus believe the measure is a relatively complete set of cognitive-appraisal processes. It is, however, possible that specific study populations warrant item modification or development so piloting the selected QOLAP_v2_ SF items would be warranted. Finally, if a full study of the “three R’s” of response shift is not a primary focus, one might select only a subset of the QOLAP_v2_ domains.

Information on QOL appraisal can be analyzed either at the item level [17] or with component scores derived from sample-specific principal components analysis [3, 16, 28]. Accordingly, such a discretionary approach to short-form creation is compatible with this general analytic paradigm. Since this approach is not standard procedure for patient-reported outcomes in general, an example of how one might go about making such a short form seems warranted.

By way of example, for a nationwide longitudinal study of healthy and chronically-ill people, we used a QOLAP_v2_ short form comprised of six Goal-Delineation items that sampled across life areas of work, practical matters, healthcare, mood, independence, and new challenges. We included four Sampling-of-Experience items that performed well both cross-sectionally and over time using similar analyses on a patient sample similar to those presented herein. We included all the Standards-of-Comparison items due to experience in a number of patient populations where the items explained clinical differences between known groups [19, 42]. Finally, we opted to include all the Combinatory-Algorithm items because the study investigated coping with a difficult and unpredictable situation, and all of the Combinatory Algorithm items seemed pertinent. This 28-item short form was viable given the good performance of the items in the QOLAP_v2_ and covered the content that was hypothesized to be relevant to the study aims.

This study has advantages in terms of a robust sample size and a participant sample that is heterogeneous in terms of chronic medical condition and many demographic variables. Its limitations should, however, be acknowledged. First, the sample is less representative of non-Whites and of males, which may affect the generalizability of our findings. Second, since the sample specifically comprises medically-ill people and their caregivers, most of whom also have medical conditions, the generalizability of our findings to people with no health problems is unknown. Future research might replicate these analyses in a healthy comparison group sampled to represent national breakdowns in terms of gender and race, as well as other demographic characteristics.

## Conclusions

In summary, results of the present study support the informativeness of the vast majority of QOLAP_v2_ items. Based on this evidence base, we suggest that investigators select the most relevant items within each domain for their ‘discretionary’ short-form measure of QOL appraisal. By retaining the domain distinctions, they will be able to characterize the different aspects of response shift in their longitudinal study. It is also our hope that this study provides not only a methodological ‘template’ for short-form development of other idiometric measures, but also a different way of conceptualizing and characterizing ‘item banks’ for such measures.

## Supplementary Information

**Additional file 1 **: **Supplemental Table 1**. Descriptive Statistics for Apprasial Items and QOL Outcomes. **Supplemental Table 2**. Results of Preliminary MANOVA Comparisons^φ^. **Supplemental Table 3**. Results of Preliminary Squared Correlation Coefficients at Baseline*. **Supplemental Table 4.** 95% Confidence Intervals for Variance Explained.

## Data Availability

The data used in these secondary analyses are confidential and thus not able to be shared.

## Abbreviations

ADL: Activities of daily living
BAI: Brief Appraisal Inventory
ES: Effect size
HIPAA: Health Insurance Portability and Accountability Act
MANCOVA: Multivariate analysis of covariance
PROMIS: Patient-Reported Outcome Measurement Information System
QOL: Quality of life
QOLAP: Quality of Life Appraisal Profile
QOLAP_v2_: QOLAP version 2
SD: Standard deviation
