# Supplementary material for: Creating idiometric short-form measures of cognitive appraisal: balancing theory and pragmatics
Source: J Patient Rep Outcomes. 2021 Jul 13;5:57. doi: 10.1186/s41687-021-00317-x (PMC8276902; doi:10.1186/s41687-021-00317-x)
Supplement: Supplementary file 1 — Additional file 1 : Supplemental Table 1. Descriptive Statistics for Apprasial Items and QOL Outcomes. Supplemental Table 2. Results of Preliminary MANOVA Comparisonsφ. Supplemental Table 3. Results of Preliminary Squared Correlation Coefficients at Baseline*. Supplemental Table 4. 95% Confidence Intervals for Variance Explained. [file 41687_2021_317_MOESM1_ESM.pdf]

Supplemental Table 1. Descriptive Statistics for Apprasial Items and QOL Outcomes

Appraisal Items	Mean	Std. Deviation	Minimum	Maximum	Skewness
**QOL Definition**					
Worry-free	3.58	0.95	1	5	-0.28
Accomplish job	3.1	1.16	1	5	0.33
Well-off	3.59	0.93	1	5	-0.1
Perfect health	3.42	0.96	1	5	0.02
Living for today	3.83	1.05	1	5	-0.57
Family time	4.27	0.93	1	5	-1.23
Not rely on others	3.5	1.14	1	5	-0.32
Legacy	2.72	1.02	1	5	0.66
Do for others	3.84	0.88	1	5	-0.4
Healthy lifestyle	3.94	0.88	1	5	-0.4
Faith	3.36	1.25	1	5	-0.06
Calm	4.05	0.85	1	5	-0.48
Independence	4.11	0.9	1	5	-0.79
No regret	3.59	1.04	1	5	-0.23
Children's successes	3.63	1.21	1	5	-0.31
Romance	3.72	1.04	1	5	-0.37
Leisure	3.91	0.88	1	5	-0.48
Responsibilities	4.21	0.8	1	5	-0.88
Family problems	3.41	1.04	1	5	-0.01
Accepting myself	4.23	0.92	1	5	-1.06
**Goal Delineation Items**					
Living conditions	2.58	1.09	1	5	0.8
Keeping up at work	2.73	1.11	1	5	0.63
Unfinished business	3	1.11	1	5	0.34
Prepare loved ones	3.71	1.14	1	5	-0.4
Resolve practical problems	3.28	1.27	1	5	0.01
Participate in upcoming	3.93	0.99	1	5	-0.65
Accomplish job	2.75	1.09	1	5	0.8
Stay in current home	3.33	1.41	1	5	-0.28
Free of regrets	3.53	1.12	1	5	-0.18
Help with health	3.22	1.19	1	5	0.14
Improve relationship	3.42	1.21	1	5	-0.11
Spiritual growth	2.98	1.28	1	5	0.27
Community contributions	2.9	0.98	1	5	0.33
Resolve money problems	3.41	1.19	1	5	-0.03
Balance	3.55	1.04	1	5	-0.14
Improve mood	3.44	1.15	1	5	-0.09
Family conflicts	2.89	1.13	1	5	0.6
Romance	2.73	1.15	1	5	0.79
Continue to drive	4.11	1.17	1	5	-1.01
Break from responsibility	2.67	1.12	1	5	0.6
Live with discomfort	3	1.31	1	5	0.07
Reduce help	2.74	1.11	1	5	0.83
Get out of rut	3.15	1.15	1	5	0.26
Solve healthcare problems	2.73	1.15	1	5	0.79
Improve health	3.73	1.01	1	5	-0.29
Role problems	2.9	1.09	1	5	0.62
Maintain healthcare	3.22	1.28	1	5	-0.17

Supplemental Table 1. Descriptive Statistics for Apprasial Items and QOL Outcomes					
**Appraisal Items**	**Mean**	**Std. Deviation**	**Minimum**	**Maximum**	**Skewness**
Keep up activiities	4.09	0.91	1	5	-0.74
Accept others	3.39	1.14	1	5	-0.04
Feel settled	3.15	1.15	1	5	0.24
Reduce doctor time	3	1.09	1	5	0.47
Let go of expectations	3.04	1.19	1	5	0.09
Stop house care	2.08	1.02	1	5	1.19
**Combinatory Algorithm Item**					
Negatives more important	2.23	1.26	1	5	0.62
Determined by others	2.6	1.36	1	5	0.26
Things beter	3.5	1.09	1	5	-0.5
Gotton used to	3.48	1.09	1	5	-0.7
Ups and downs	3.05	1.25	1	5	-0.19
Keep up mood	3.7	1.15	1	5	-0.74
Recent events	3.3	1.27	1	5	-0.41
Obligations not accomplishments	3.54	1.18	1	5	-0.63
Recent changes	3.24	1.23	1	5	-0.32
**Sample of Experience Item**					
Worst moments	2.68	0.98	1	5	0.15
Emphasize positive	3.85	0.94	1	5	-0.61
Recent few weeks	3.55	0.97	1	5	-0.56
Relevant past 3 mo.	3.2	1.11	1	5	-0.23
Balance positive/negative	3.63	1.03	1	5	-0.52
Recent flare-ups	3.01	1.16	1	5	-0.08
Future	3.94	0.99	1	5	-0.88
Focus on health	3.63	1.11	1	5	-0.55
Relationships	4.06	0.89	1	5	-0.97
Doctor told	3.22	1.23	1	5	-0.25
Only for survey	2.76	1.15	1	5	-0.02
First reaction	4.06	0.94	1	5	-1.14
Not complain	3.74	1.12	1	5	-0.75
Seriousness	3.41	1.15	1	5	-0.38
**Standards of Comparison Item**					
Others with same condition	2.33	1.21	1	5	0.43
Healthy others	2.79	1.28	1	5	0.02
Doctor said	2.44	1.17	1	5	0.29
Perfect health	3.02	1.27	1	5	-0.12
Life working for	3.49	1.14	1	5	-0.63
Way others see you	2.97	1.23	1	5	-0.14
People your age	2.99	1.25	1	5	-0.19
Time before health condition	2.9	1.34	1	5	-0.03
Family treated for same health condition	2.17	1.3	1	5	0.69
**QOL Scores**					
Change in ADL	0.35	9.49	-30.00	30.00	0.07
Change in PROMIS Physical Health	-3.81	11.31	-57.70	32.80	-0.92
Change in PROMIS Mental Health	-0.40	8.03	-62.50	40.50	-1.47

Supplemental Table 2. Results of Preliminary MANOVA Comparisons[ψ]

Domain	Abbreviated Item	Eta Squared*				
		Region	Gender	Whether Working	Whether Retired	Whether Disabled
**QOL Definition**						
	Accepting myself	0.00	0.00	0.00	0.00	0.00
	Accomplishing at job	0.01	0.00	0.13	0.01	0.00
	Do for others	0.00	0.00	0.00	0.00	0.00
	Do things independently	0.01	0.00	0.00	0.01	0.00
	Perfect health	0.01	0.00	0.00	0.00	0.01
	Rid of family problems	0.01	0.00	0.00	0.00	0.00
	Well-off financially	0.01	0.00	0.00	0.00	0.00
	Worry-free	0.01	0.00	0.00	0.00	0.00
	Calm and peaceful	0.01	0.00	0.00	0.00	0.00
	Healthy lifestyle	0.01	0.00	0.00	0.00	0.00
	Leisure time	0.01	0.00	0.01	0.00	0.00
	Love and romance	0.01	0.01	0.00	0.01	0.01
	Nothing to regret	0.01	0.00	0.00	0.00	0.00
	Living for today	0.00	0.00	0.00	0.00	0.00
	Teachings of my faith	0.04	0.00	0.00	0.00	0.00
	Legacy	0.01	0.00	0.00	0.00	0.00
	Not relying on others	0.01	0.00	0.00	0.00	0.00
	Children's successes	0.00	0.00	0.00	0.01	0.01
	Time with family	0.00	0.00	0.00	0.00	0.00
	Responsibilities	0.01	0.00	0.00	0.00	0.00
**Goal Delineation**						
	Keeping up at work, school	0.01	0.00	0.01	0.02	0.01
	Contributions to the community	0.00	0.00	0.00	0.00	0.00
	Growing as a spiritual person	0.03	0.00	0.00	0.00	0.00
	Accept people as they are	0.01	0.01	0.00	0.00	0.00
	Break from being responsible	0.01	0.01	0.00	0.00	0.00
	Loved ones to be prepared	0.01	0.01	0.00	0.01	0.01
	Participate fully in upcoming events	0.01	0.00	0.00	0.00	0.00
	Free of regrets	0.00	0.00	0.00	0.00	0.00
	Reduce the time on health issues	0.01	0.00	0.01	0.00	0.00
	Continue to drive	0.00	0.00	0.00	0.01	0.00
	Feel more settled about situations	0.02	0.00	0.00	0.01	0.00
	Find love and romance	0.01	0.00	0.00	0.00	0.00
	Do things to improve my health	0.01	0.00	0.00	0.00	0.00
	Balance between obligations and leisure activities	0.01	0.01	0.00	0.02	0.00
	Get more help from doctors	0.01	0.00	0.01	0.01	0.01

Domain	Abbreviated Item	Region	Gender	Whether Working	Whether Retired	Whether Disabled
	Get out of a rut	0.01	0.00	0.01	0.01	0.00
	Feel more content about circumstances	0.01	0.00	0.00	0.02	0.00
	Improve important relationship	0.00	0.00	0.00	0.01	0.00
	Keep up with activities	0.01	0.00	0.00	0.00	0.00
	Live with discomfort	0.01	0.01	0.00	0.00	0.01
	Let go of expectations	0.01	0.01	0.00	0.00	0.00
	Reduce the help needed	0.01	0.00	0.00	0.00	0.02
	Resolve conflicts	0.00	0.00	0.00	0.01	0.00
	Resolve money problems	0.00	0.00	0.00	0.02	0.00
	Solve role problems	0.01	0.00	0.00	0.02	0.00
	Solve healthcare problems	0.01	0.00	0.00	0.00	0.00
	Stay in my current home	0.01	0.00	0.00	0.02	0.00
	Stop having to take care of a home	0.01	0.00	0.00	0.00	0.00
	Take care of unfinished business	0.01	0.00	0.00	0.00	0.00
	Maintain current medical and health care	0.01	0.00	0.00	0.01	0.00
	Get used to living conditions	0.01	0.00	0.00	0.00	0.00
	Resolve specific practical problem	0.00	0.00	0.00	0.01	0.00
	There is much more I want to accomplish at my job	0.00	0.00	0.05	0.01	0.01
**Sampling of Experience**						
	Balance the positives with the negatives	0.01	0.01	0.00	0.00	0.00
	Only think about for a questionnaire like this	0.01	0.00	0.00	0.00	0.00
	Relationships with family and friends	0.01	0.00	0.00	0.00	0.00
	Emphasize the positive	0.01	0.01	0.00	0.01	0.00
	Thinking about the worst moments	0.01	0.00	0.00	0.01	0.00
	Focus on your health condition	0.01	0.00	0.00	0.00	0.01
	Recall recent	0.01	0.00	0.00	0.00	0.00
	Doctor told you	0.00	0.00	0.00	0.01	0.01
	Future	0.00	0.00	0.00	0.00	0.00
	Things going recently	0.01	0.00	0.00	0.00	0.00
	Try not to complain	0.01	0.00	0.00	0.00	0.00
	Communicate the seriousness of the situation	0.01	0.00	0.00	0.00	0.01
	First reaction	0.01	0.00	0.00	0.00	0.00
	Remember everything relevant	0.01	0.00	0.00	0.00	0.00

** Highlighted text indicates an Eta-squared reflecting at least a small effect size.*

[ψ] *We included this subset of three out of five domains as an adequate number for these exploratory tests .*

Supplemental Table 3. Results of Preliminary Squared Correlation Coefficients at Baseline*

Domain	Abbreviated Item	Age	No. Comorbidities	Bills
**QOL Definition**				
	Accepting myself	0.00	0.00	0.00
	Accomplishing at job	0.03	0.02	0.01
	Do for others	0.00	0.00	0.00
	Do things independently	0.01	0.00	0.00
	Perfect health	0.01	0.02	0.00
	Rid of family problems	0.00	0.00	0.01
	Well-off financially	0.00	0.00	0.00
	Worry-free	0.00	0.00	0.00
	Calm and peaceful	0.00	0.00	0.00
	Healthy lifestyle	0.00	0.00	0.00
	Leisure time	0.00	0.00	0.01
	Love and romance	0.02	0.02	0.01
	Nothing to regret	0.00	0.00	0.00
	Living for today	0.00	0.00	0.00
	Teachings of my faith	0.01	0.00	0.00
	Legacy	0.00	0.00	0.00
	Not relying on others	0.00	0.00	0.01
	Children's successes	0.00	0.00	0.00
	Time with family	0.01	0.01	0.00
	Responsibilities	0.00	0.00	0.00
**Goal Delineation**				
	Keeping up at work, school	0.05	0.00	0.00
	Contributions to the community	0.00	0.00	0.00
	Growing as a spiritual person	0.01	0.00	0.00
	Accept people as they are	0.00	0.01	0.00
	Break from being responsible	0.00	0.03	0.02
	Loved ones to be prepared	0.01	0.03	0.01
	Participate fully in upcoming events	0.00	0.00	0.00
	Free of regrets	0.00	0.00	0.00
	Reduce the time on health issues	0.00	0.05	0.02
	Continue to drive	0.02	0.00	0.00
	Feel more settled about situations	0.01	0.00	0.02
	Find love and romance	0.01	0.00	0.01
	Do things to improve my health	0.00	0.02	0.01
	Balance between obligations and	0.02	0.00	0.02
	Get more help from doctors	0.00	0.08	0.05
	Get out of a rut	0.01	0.03	0.05

Supplemental Table 3. Results of Preliminary Squared Correlation Coefficients at Baseline*

Domain	Abbreviated Item	Age	No. Comorbidities	Bills
	Feel more content about	0.02	0.03	0.04
	Improve important relationship	0.00	0.01	0.01
	Keep up with activities	0.00	0.00	0.00
	Live with discomfort	0.01	0.05	0.01
	Let go of expectations	0.00	0.03	0.01
	Reduce the help needed	0.01	0.04	0.04
	Resolve conflicts	0.00	0.02	0.02
	Resolve money problems	0.02	0.03	0.19
	Solve role problems	0.03	0.01	0.02
	Solve healthcare problems	0.00	0.05	0.05
	Stay in my current home	0.06	0.00	0.00
	Stop having to take care of a home	0.00	0.01	0.02
	Take care of unfinished business	0.00	0.02	0.02
	Maintain current medical and health	0.03	0.00	0.00
	Get used to living conditions	0.00	0.02	0.03
	Resolve specific practical problem	0.00	0.04	0.13
	There is much more I want to	0.05	0.01	0.00
**Sampling of Experience**				
	Balance the positives with the	0.00	0.01	0.02
	Only think about for a questionnaire	0.00	0.00	0.00
	Relationships with family and friends	0.00	0.00	0.00
	Emphasize the positive	0.02	0.01	0.02
	Thinking about the worst moments	0.01	0.03	0.04
	Focus on your health condition	0.00	0.07	0.01
	Recall recent	0.02	0.05	0.03
	Doctor told you	0.02	0.04	0.00
	Future	0.00	0.00	0.00
	Things going recently	0.00	0.00	0.00
	Try not to complain	0.00	0.00	0.00
	Communicate the seriousness of the	0.00	0.02	0.01
	First reaction	0.00	0.00	0.00
	Remember everything relevant	0.00	0.01	0.01

**More saturated colors reflect larger effect sizes.*

Supplemental Table 4.
95% Confidence Intervals for Variance Explained

Variance Explained*	Frequency	95% Confidence Interval**	
		Lower Limit	Upper Limit
0.01	250	0.00	0.02
0.02	217	0.01	0.03
0.03	65	0.01	0.05
0.04	51	0.02	0.06
0.05	25	0.03	0.07
0.06	30	0.04	0.08
0.07	24	0.04	0.10
0.08	24	0.05	0.11
0.09	170	0.06	0.12
0.10	25	0.07	0.13
0.11	6	0.08	0.14
0.12	5	0.09	0.15
0.13	3	0.09	0.17
0.14	2	0.10	0.18
0.15	3	0.11	0.19
0.17	2	0.13	0.21
0.18	1	0.14	0.22
0.23	3	0.19	0.27
0.24	3	0.20	0.28
0.37	3	0.32	0.42

** When rounded to two decimal places. Values that rounded to 0.00 are excluded.*

***With N = 1,391, variations in number of model predictors (7 <= k <= 9) do not change these values even at 3 decimal places.*
